# Direct evidence for eudicot pollen-feeding in a Cretaceous stinging wasp (Angiospermae; Hymenoptera, Aculeata) preserved in Burmese amber

**DOI:** 10.1038/s42003-019-0652-7

**Published:** 2019-11-07

**Authors:** David A. Grimaldi, Enrique Peñalver, Eduardo Barrón, Hollister W. Herhold, Michael S. Engel

**Affiliations:** 1 American Museum of Natural History, Central Park West at 79th Street, New York, NY 10024-5192 USA; 2 Museo Geominero, Instituto Geológico y Minero de España. Ríos Rosas 23, E-28003 Madrid, Spain; 3 Division of Entomology, Natural History Museum, and Department of Ecology and Evolutionary Biology, University of Kansas, Lawrence, Kansas 66045 USA

**Keywords:** Evolution, Ecology

## Abstract

Angiosperms and their insect pollinators form a foundational symbiosis, evidence for which from the Cretaceous is mostly indirect, based on fossils of insect taxa that today are anthophilous, and of fossil insects and flowers that have apparent anthophilous and entomophilous specializations, respectively. We present exceptional direct evidence preserved in mid-Cretaceous Burmese amber, 100 mya, for feeding on pollen in the eudicot genus *Tricolporoidites* by a basal new aculeate wasp, *Prosphex anthophilos*, gen. et sp. nov., in the lineage that contains the ants, bees, and other stinging wasps. Plume of hundreds of pollen grains wafts from its mouth and an apparent pollen mass was detected by micro-CT in the buccal cavity: clear evidence that the wasp was foraging on the pollen. Eudicots today comprise nearly three-quarters of all angiosperm species. *Prosphex* feeding on *Tricolporoidites* supports the hypothesis that relatively small, generalized insect anthophiles were important pollinators of early angiosperms.

## Abstract (web summary)

Grimaldi et al. report on a primitive stinging wasp preserved in mid-Cretaceous amber from Myanmar, encapsulated just after it fed on the pollen of a eudicot. Micro-CT shows a pollen mass in the wasp’s mouth cavity, supporting the idea that insect anthophiles were important pollinators of early angiosperms.

## Introduction

Among symbiotic relationships unique to land, such as between fungi and plants in the forms of lichens and mycorrhizae, the pollination of angiosperms by insects has special ecological significance. Some 80–95% of the ~295,000 species of angiosperms are pollinated by insects (the proportions vary with ecosystem^1,2^), a species diversity that is commonly explained as a result of this symbiosis^3^. Besides promoting heterozygosity and sexual recombination, insect pollination confers critical ecological benefits, by allowing reproduction among distant plants. Dispersed plants can better exploit limiting resources such as light gaps, moisture, and nitrogen, and they have reduced exposure to diseases and defoliating insects that overwhelm dense monocultures^4^. It is likely, in fact, that insect pollination (entomophily) is the ancestral condition among angiosperms. Some gnetaleans such as *Welwitschia* and *Gnetum*, close gymnosperm relatives of angiosperms, are pollinated by assorted flies and beetles^5^, as are the phylogenetically basal Amborella, Nymphaeales, Illiciaceae, Trimeniaceae, and Austrobaileyales (ANITA) grade of angiosperm families^6,7^.

The paleontological record is gradually yielding data on the co-occurrence of insects with plant reproductive structures in geological time, providing evidence that is both direct (e.g., a fossil insect with pollen) or inferential (e.g., fossilized floral or foraging structures specialized for entomophily). The most overt insect structure specialized for anthophily is a long proboscis. In this condition the insect mouthpart appendages are extended for reaching into plant reproductive structures for feeding on nectar and pollen, having evolved multiple times among insects in various forms^4^. The discovery of diverse, long-tongued Mesozoic insects has revealed an unexpected array of specialized early anthophiles^8^. Although a long proboscis is correlated with anthophily (though not perfectly), the mouthparts in most groups of anthophilous insects in fact are not modified as such; many species are behaviorally specialized. For example, with the exception of the mostly wind-pollinated conifers, basal seed plants (including the ANITA grade of angiosperms) attract small, generalized beetles (e.g., staphylinids and scarabs), Diptera (sciaroid and culicomorphan midges; lauxaniid, ephydrid, and calliphorid flies), and short-tongued halictid bees^5–7^. Many large genera of bees, such as *Andrena*, *Megachile*, and *Perdita*, are morphologically generalized but oligolectic (specializing in feeding on a particular genus or family of angiosperms). Thus, the fossil record of long proboscides may greatly underestimate the extent of early anthophily.

Caution is also required when inferring an insect diet on the basis of just a long proboscis. For example, Early Cretaceous nemestrinid and tabanomorph flies with long proboscides were interpreted as angiosperm pollinators^9^, but a zhangsolvid fly in Early Cretaceous Spanish amber—with an even longer proboscis—had a pollen load from a gymnosperm in the Mesozoic group Benettitales^10^. It is possible that these Cretaceous long-tongued flies may have been feeding on early angiosperms as well, but clearly they were not restricted to them. Also, mouthpart structures presumed to be adaptations for floral feeding may well be exaptations that originally arose for other functions, even those predating the appearance of flowering plants. Species of Mesozoic scorpionflies in and related to the family Pseudopolycentropodidae, have long proboscides, apparent perfect fits for probing the narrow pollen tubes of extinct gymnosperms^11^. The unusual, dipterous *Parapolycentropus* in Burmese amber, which has a fine, stylet-like proboscis, shares adaptive features with many empidid and ceratopogonid flies that today are insectivorous, a diet typical of mecopterans^12^, but a specimen of this scorpionfly was recently found with nearby *Cycadopites* pollen^13^. It may have actually fed on both: some hematophagous species of mosquitoes also feed on nectar and are effective pollinators^14^. Pollen on or in the fossil insect provides definitive, direct evidence of diet.

In some reports on Cretaceous insects, the associated pollen was interpreted to be from possible or stem-group angiosperms, but which are actually gymnosperms. The first such reports concern pollen in the digestive tracts of lithified xyelid sawflies from the Early Cretaceous of Baissa, Siberia (Zaza Formation: Hauterivian-Barremian)^15,16^. Xyelidae are a small, extant Holarctic family of 82 species, the basal-most one in the Hymenoptera, whose fossil record extends to the Triassic. Larvae and adults of modern species feed extensively on the staminate cones of pines (*Pinus* spp.); adult mouthparts are well adapted for grazing on this and even some angiosperm pollen^17^. Three of the fossil sawfly species (*Anthoxyela anthophaga*, *Spatoxyela pinicola*, and *Ceroxyela dolichocera*) contained bisaccate and bilobed-monosaccate pollen grains from different species of conifers^15,18^. *Spathoxyella* contained pollen from the extinct gnetalean *Baisanthus*. Another fossil sawfly contained sulcate pollen similar to *Eucommiidites* (Erdtmanithecales)^16^, reported as *Cryptosacciferites* and a possible stem-group angiosperm. Even though *Eucommiidites* has three colpi as in angiosperms, the massive tectum and alveolate exines of pollen from the Baissa wasp indicate it is gymnosperm (the grains lack the rod-like columellae and roof-like tectum typical of angiosperms). All of these pollen species are abundant in Eurasian Cretaceous strata^19,20^, which, with their bisaccate structure, are features of wind-transported pollen^21^. Another insect–pollen relationship from the famous Baissa outcrops involves *Classopolis* pollen (belonging to the extinct conifer family Cheirolepidiaceae) on a lithified brachyceran fly attributed to Asilomorpha^22^.

Three instances of Cretaceous insects carrying pollen are in Albian-aged amber from Spain, all involving gymnosperms. One concerns a genus of thrips (order Thysanoptera: family Melanthripidae) with specialized setae apparently specialized for collecting pollen^23^. Some modern thrips feed on anthophyte pollen, including melanthripids; the one in Spanish amber carried pollen of *Cycadopites*, which is probably Cycadalean. The second case is a basal brachyceran fly in the extinct family Zhangsolvidae, *Buccinatormyia magnifica*, with a long, rigid proboscis, found with a clump of *Exesipollenites* pollen adhering to its body^10^. *Exesipollenites* is a gymnosperm probably within the extinct group Bennettitales. The third case concerns an oedemerid beetle preserved with cycad pollen on its body^24^.

Interestingly, another family of beetles (Boganiidae) has also been found with cycad pollen^25^, but in amber from the mid-Cretaceous of northern Myanmar, the most diverse Cretaceous deposit in the world and which is steadily yielding other direct insect–pollen evidence, including our present report. A general report on Burmese amber presented good photographic evidence for a permopsocid (small, stem-group relatives of living bark lice and other Psocodea), which has definitive tricolpate pollen in its gut^26^, but little further study or discussion of this specimen has been made.

Two reports of Cretaceous insects with pollen are difficult to evaluate. One of these was another xyelid sawfly, but in Aptian-aged limestone from the Crato Formation of Brazil^27^. The pollen in this xyelid was identified as *Afropollis*, a widespread Cretaceous genus of pollen that is spheroidal, reticulate, acolumellate, and with a loose reticulum, putatively in or close to the basal angiosperm families Winteraceae and Schizandraceae^28,29^. However, ultrastructural studies of its exine support *Afropollis* having been produced by a non-angiosperm anthophyte^30^. Also, this original report^27^ was unfortunately a meeting abstract without images or other documentation, and efforts by one of us (D.G.) to find this specimen in Brazil failed, so the identity of the pollen is impossible to confirm. The other report^31^ concerns a fly in Burmese amber that is a stem-group bibionid, a family that today facultatively visits flowers. Photographs of the putative pollen lack detail necessary to determine whether it is angiosperm, even though the author attributed the minute grains to two species of flowers in Burmese amber, based on overlap of the grain size and shape^31^.

Here we report an exceptional discovery from the Cretaceous record in which definitive angiosperm pollen is preserved with a pollen feeder and possible pollinator. It is one of just a few records of such an association where the pollen is unquestionably angiosperm, in fact belonging to a large, derived lineage of angiosperms, the eudicots. This is also the only such Cretaceous record involving a wasp in the Aculeata (stinging wasps), the major group of insect pollinators that includes the bees. As we discuss later, various aspects of aculeate structure, behavior, and biology make these insects probably the most effective insect vectors of pollen.

## Results

**The wasp**

Family Incertae Sedis

***Prosphex*** **Grimaldi and Engel, new genus**

**ZooBank LSID:** urn:lsid:zoobank.org:act:1F27660C-5025-479E-8C4F-F7C10600EF24

**Fig. 1:**
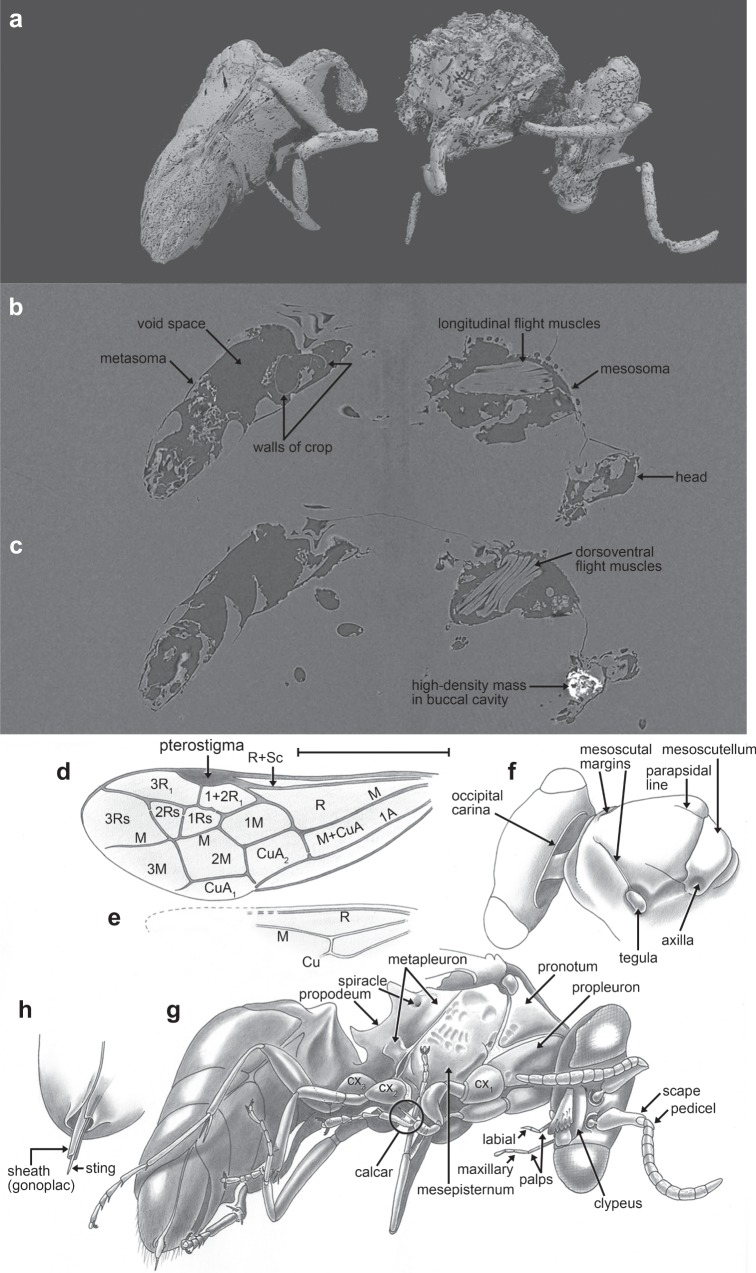
CT images and illustrations of the holotype of *P. anthophilos*, new genus, new species, AMNH Bu-KL18-31. **a** CT image, external surface. Portions of thin and/or distorted cuticle are missing, particularly in the mid-section. **b**, **c** Two CT slices through (**a**), showing the longitudinal (**b**) and dorsoventral flight muscles (**c**), walls of the crop (**b**), and a high-density mass in the buccal cavity (**c**), likely a pollen mass that is partially pyritized. **d**–**h** Illustrated rendering of *Prosphex*, showing the forewing (**d**, with conventional abbreviations for wing veins and cells) and the hind wing (**e**) (slightly reconstructed), dorsal view of head and thorax (**f**), left ventrolateral habitus (**g:** cx, coxa) and detail of sting (**h**). Body of the wasp is rendered as preserved, without reconstruction. All images to the same scale (scale line 1.0 mm); **h** is slightly magnified

**Diagnosis:** A medium-sized aculeate (body length ca. 4.3 mm, excluding antennae), body compact, with scattered sparse simple setae, where evident such setae minute (Fig. 1 and Supplementary Figs. 1, 2); macropterous, with forewing venation complete and generally plesiomorphic, with 14 well-defined cells, apices of M and Cu nearly reaching wing margin (Fig. 1d and Supplementary Fig. 3b); basal vein gently arched, slightly distad 1cu-a; R extending along the wing margin beyond marginal cell to the wing apex, marginal cell apex acutely rounded on the anterior wing margin; third submarginal cell broader anteriorly than posteriorly; 1m-cu entering second submarginal cell in proximal quarter; 2m-cu slightly basad 2rs-m, nearly confluent; antenna with 11 flagellomeres, antennal toruli low on face, meeting epistomal sulcus (no subantennal area); compound eyes bare; ocelli either absent (highly unusual) or small and obscure; occipital carina present, pronotal lobe lacking, posterolateral angle of pronotum angulate and extending posteriorly to meet the tegula; mesoscutal sulci reduced to parapsidal lines and incomplete mesoscutal margins, notauli apparently absent; femora not crassate (although metafemur very slightly broader than pro- and mesofemora); tibiae slender and cylindrical; tibial spur formula 1-2-2, metatibial spurs simple; metabasitibial plate absent; pretarsal claws with minute inner tooth, arolium present, and small; propodeum broad (plesiomorphically similar to chrysidoids).

**Type Species**: *P. anthophilos* Grimaldi and Engel, new species.

**Etymology**: Greek (masculine), *pro*- (first, before), and -*sphex* (wasp, a common suffix for aculeate wasp genera), in reference to the plesiomorphic nature and mid-Cretaceous age of the genus.

**Comments**: The sting, loss of cerci, and antennal structure (large scape and 11 short, stout flagellomeres) indicate that *Prosphex* is clearly an aculeate wasp. There are diverse aculeates belonging to ~15 living and extinct families preserved in Burmese amber^32,33^. *Prosphex* is distinctively plesiomorphic and does not belong to any of the three main lineages of aculeates as defined on the basis of their modern representatives (Chrysidoidea, Apoidea, and Vespoidea)^34,35^. The forewing venation is plesiomorphically nearly complete, with even the apices of forewing veins M and CuA_1_ virtually but not quite reaching the wing margin (Fig. 1d), unlike any living or fossil chrysidoids. Furthermore, the propodeum-metapleural suture appears to be absent, a plesiomorphic feature that would place the current fossil outside of Chrysidoidea. The lack of pronotal lobes excludes *Prosphex* from the Apoidea (which includes bees, sphecid, crabronid, and other wasps). The presence of 11 flagellomeres in the female excludes *Prosphex* from the Vespoidea or Apoidea, females of both having 10 flagellomeres (males have 11). The complete wing venation is easily derived from the earliest aculeates, such as Bethylonymidae (a possibly paraphyletic or even polyphyletic group lithified in the Late Jurassic of Kazakhstan) (Figs. 106, 107, 109, and 110 in ref. ^36^), but those wasps have, e.g., a strong mesoscuto-mesoscutellar sulcus in the middle of the mesosoma. *Prosphex* has very reduced sulci and the mesoscutellum is much smaller than the mesoscutum. *Prosphex* appears to be representative of a stem-group lineage that diverged prior to the divergence of the three main lineages of aculeates, although it is uncertain whether the genus could instead be a stem group to chrysidoids, sister to all Aculeata, or even sister to Euaculeata. The eventual discovery of the male and further material would greatly elucidate the phylogenetic placement of this otherwise plesiomorphic wasp.

***P. anthophilos*** **Grimaldi and Engel, new species**

**Diagnosis**: As for genus, by monotypy.

**Etymology**: Greek (masculine), *antho* (flower or pollen), and -*philos* (loving), in reference to its preserved pollen meal.

**Description**: See Supplementary Information.

**Holotype**: AMNH Bu-KL18-31, from approximately the Albian–Cenomanian boundary of Kachin Province, northern Myanmar; deposited in the American Museum of Natural History, New York.

### The pollen

**Fig. 2:**
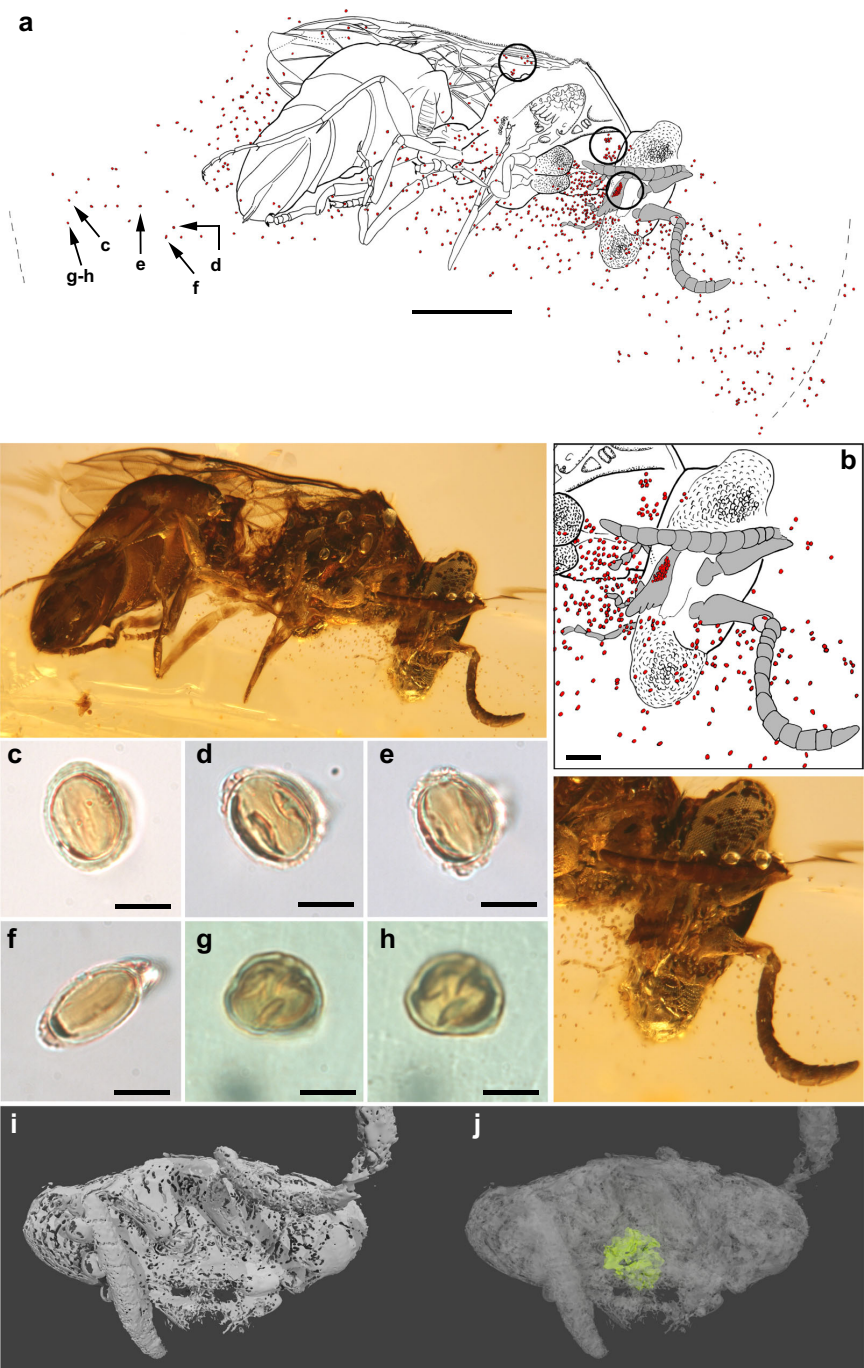
Angiosperm pollen load of the wasp, *P. anthophilos*, and pollen grain features (*Tricolporoidites* sp.). **a** Map of the preserved pollen grains in contact with (encircled) and surrounding the wasp, and microphotograph of the wasp at the same scale. Scale line 1.0 mm. The grains labeled **c**–**h** refer to those shown at high magnification in Fig. 2c−h. **b** Detail of the concentrated pollen mass on and around the mouthparts (drawing and microphotograph from **a**; at the same scale). Scale line 0.2 mm. **c**–**e** Subprolate pollen grains in equatorial view (note the colpi with poroids). **f** Prolate and colporoidate pollen grain in equatorial view. **g**, **h** Subprolate pollen grain in polar view showing its three colpi (same grain with different focus). Microphotographs **c**–**h** taken using a ×100 objective and at the same scale. Scale lines in **c**–**h**: 10 μm. **i**, **j** High-resolution CT scans of the wasp head (frontal view), showing just the external surface (**i**) and with the external surface faded to reveal the food mass in the buccal cavity (in green) (**j**)

The visible pollen load associated with the fossil wasp contains 656 mapped grains plus a mass of an indeterminate number of grains behind the right mandible and in the buccal cavity (Fig. 2a, b, j and Supplementary Figs. 1–3). This is a minimal estimate, because the number of grains in contact with the body are obscure (there are at least 30) and some were lost when the piece was originally ground and polished after excavation (some grains were exposed at the amber surface; as a result, a few are located micrometers from the surface, allowing observation with a ×100 oil-immersion objective [total ×1000 magnification] [Fig. 2c–h]). Visible grains in contact with the body occur on the right mandible, the prosternum close to the right mandible, and bases of the wings (Fig. 2a, b). Most of the pollen grains are individually scattered in plumes ventral to the body of the wasp (a few grain clusters occur), dissipating anteriorly and ventrally from their concentration near the mouthparts, the apparent source of the plumes. Most pollen grains in the plumes are badly preserved, corresponding to areas of poor preservation of the wasp, each of these grains being surrounded by a thin gas layer and sometimes deformed in the direction of the axis from the wasp. Fortunately, the pollen grains virtually in contact with the amber surface and observed at ×1000 original magnifications are finely preserved.

The pollen grains (Fig. 2c–h) are tricolporoidate, isopolar, and tectate; the polar axis 14.28–19.00 μm long, equatorial diameter 12.38–14.28 μm, the shape subprolate to prolate (P/E 1.11–1.81), apocolpia rounded. Colpi are long, straight, nearly reaching the poles. In the equatorial area, along the colpi, a somewhat thinner exine is divided, forming small and nearly circular poroids of ~1.43 μm diameter. The exine is 1–1.5 μm thick and the surface psilate to shagrinate.

These pollen grains belong to the angiosperm form genus *Tricolporoidites*, erected on the basis of pollen in early Cenomanian strata of the Bohemian Basin (Czech Republic)^37^. This form genus was emended^38^ based on specimens from the late Albian of the Cheyenne and Kiowa Formations, Kansas (USA), emphasizing an isopolar pattern. *Tricolporoidites* pollen occurs in mid-Cretaceous amber-bearing strata from Europe^39,40^. The pollen preserved with the wasp greatly resembles the species *Tricolporoidites subtilis* Pacltová^37^ (1971: p. 117, pl. 9, figs. 5–9, 17), which was described from the Upper claystones of the Louny-1 bore in the Peruc Formation (Bohemian Basin). However, the type specimens are clearly smaller in size, ranging from 11 to 13 μm in the polar axis and 10 to 12 μm in equatorial diameter. *T. subtilis* has also been identified in the early Cenomanian of the Archingeay-Les Nouillers succession (Charentes, W France)^40^. The grains of *Tricolporoidites* sp. from Brnik (Bohemian Basin), figured but not described by Pacltová^37^ (1971: pl. 9, figs. 10–13), are similar in their polar orientation to those in the wasp’s pollen load.

**Fig. 3:**
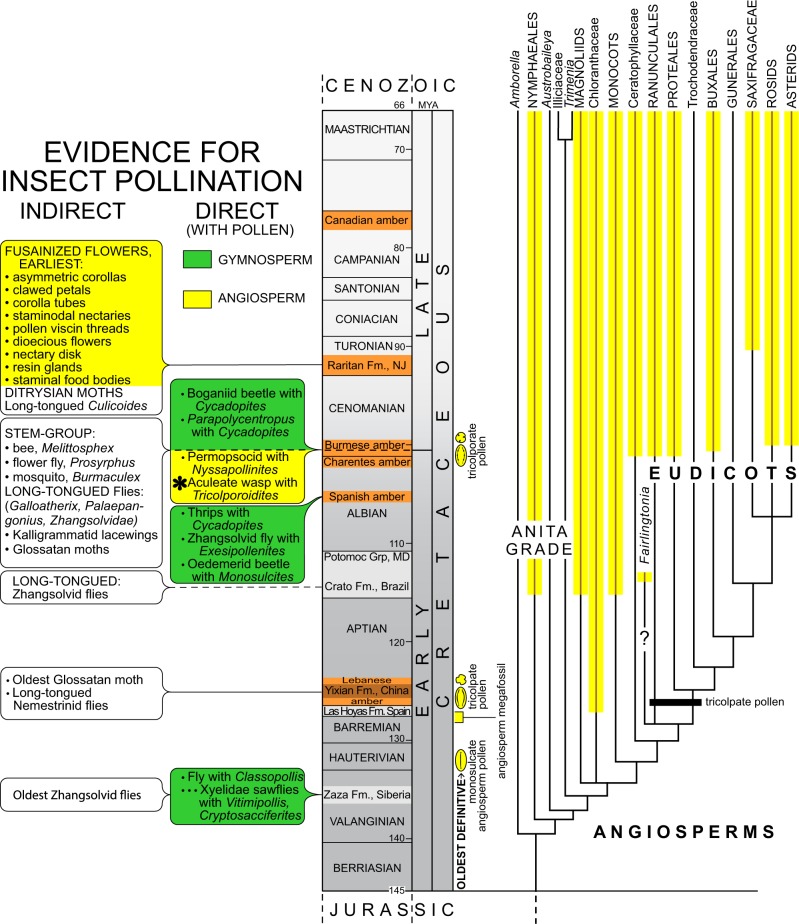
Summary diagram of records of insect–pollen association in the Cretaceous, showing on the left inferential/indirect evidence (features of fossil flowers or insects specialized for entomophily and anthophily, respectively) and direct evidence (an insect having pollen in or on the body). Fossil records are based on various references, many cited in the text^4,11,16,23,24,26,42,55,57,64^. All angiosperm records or features are depicted in yellow. Relationships among basal lineages of angiosperms are based on APG^74^; divergence times are arbitrary and are not intended to reflect modeled estimates

*Tricolporoidites* was not reported in the short list of palynomorphs from the amber-bearing sediments in northern Myanmar^41^, which is not surprising, as pollen that is dispersed by wind greatly predominates in the geological strata; entomophilous pollen is generally rare. According to Ward^38^, the botanical affinity of *Tricolporoidites* corresponds to a non-magnoliid dicot, but a source family has not yet been determined. Tricolporate pollen occurs in the core eudicots (rosids and asterids, Fig. 3) and also in some basal eudicots such as Buxaceae, Sabiaceae, and Menispermaceae^42^; tricolporoidate pollen occurs in the core eudicots.

*Tricolporoidites* pollen is distinctive and was not produced from any of the ~15 species (in 5–6 families and 5 orders) of described or undescribed angiosperms preserved as flowers in Burmese amber^32,43–46^. Approximate phylogenetic positions of some of these flowers are established, such as in the Laurales and monocots^47^ and rosids^43^, but some may be improperly attributed, such as *Eoëpigynia* being in the Cornaceae^48^; its epigynous and tetramerous flowers are features found in distantly related groups such as Saxifragales, Myrtales, and Asterales^49^. The large size and tricolporoidate apertures also distinguish *Tricolporoidites* from pollen associated with the flowers of *Lijinganthus revoluta*, attributed to the Pentapetalae in core eudicots^44^; this flower genus has tricolpate pollen. Tricolpate pollen grains of the genus *Nyssapollenites* occur within a permopsocidan insect^26^ in Burmese amber and pollen of *Eoëpigynia* is putatively tricolpate^48^ but needs to be confirmed. Most of the Burmese amber flowers are unplaced and require a detailed study.

Recently, *Tricolporoidites* has been assigned to the eudicot angiosperms^40^. Tricolpate pollen, in fact, is the single defining mophological feature of the eudicots, a radiation comprising ~72% of the living species of angiosperms^42^.

### Pollen meal

Despite compression and distortion of some portions of the wasp, especially in the propodeal region, the preservation is excellent. Computed tomography (CT) scans reveal even internal organs and tissues preserved with fidelity, the striations and positions of the dorsoventral and longitudinal flight muscles, e.g., intact and easily discerned in slice-away lateral sections, as are the walls of an apparently empty crop, which is the food storage organ (Fig. 1b, c). Three high-density areas were found inside the wasp using CT scanning (Fig. 1c). Areas of similar density were not found anywhere else in the amber. The largest high-density mass was located in the buccal cavity (Fig. 1c and Fig. 2i, j), just posterior to the mandibles, and corresponds to an area where a mass of pollen grains is partially visible behind the right mandible. Two small high-density areas are in the mesosoma, possibly within the esophagus. It is possible that these high-density areas comprised minute granules of pyrite (iron disulfide), as this mineral commonly forms in fine cracks and interstices within the amber, often infiltrating inclusions. Pyrite forms within amber, because amber-bearing sediments are typically highly reducing environments rich in sulphur and iron. However, the granularity of the high-density masses shows no cubic or otherwise geometric crystalline structure in CT scans (rather, they are rounded and amorphous Fig. 2i, j), nor does light microscopy or CT scanning reveal any fine fractures connecting these areas to the amber surface (some fine fractures occur near the wasp, but these are entirely internal, see Supplementary Fig. 2a). Another possibility is that the high-density areas are masses of pollen grains that are also nuclei for the formation of pyritic microcrystals.

Insects visit flowers for many purposes, from occasional perching and basking, to mating, an attraction to odors, adult feeding on pollen and/or nectar, floral deception and oviposition, to the gathering of pollen, nectar, essential oils or other substances for nest provisioning, or mate attraction^50^. Some of these, particularly the last three behaviors, are associated with insects that are obligate pollinators^4^. *Prosphex* doubtlessly was a pollen feeder. The concentration of pollen around the head and especially near the mouthparts, from which plumes of it dissipate (Fig. 2a), the mass in the buccal cavity (Fig. 2i, j), and the three areas where pollen grains adhere to the body, all indicate that the co-occurrence of the pollen and this wasp was not a chance encounter. The visible pollen grains show minimal differences in size and morphology not attributable to differential preservation (Fig. 2c–h), indicating that all are the same *Tricolporoidites* species, although whether it derived from a single or multiple plants is impossible to say. If additional specimens of *P. anthophilos* with pollen loads are discovered it would provide direct evidence as to how polylectic or flower constant this species was, much like the series of Early Cretaceous xyelid wasps with gymnosperm pollen meals^16^.

## Discussion

A summary of the evidence for insect pollination in the Cretaceous is provided in Fig. 3, based on both direct (i.e., an insect with pollen) and indirect evidence (i.e., insect taxa that today are pollinators, and insects with conspicuous adaptive features, such as a long proboscis). The evidence for early pollination by insects is rare, mostly indirect and inferential, despite angiosperms preserved in a geological pageant of fossil leaves, stems, wood, and flowers in rocks and amber, as well as pollen that pervades sediments.

The tradeoff in the pollen fossil record is that it is much more extensive than the record of vegetative and reproductive organs, but pollen morphology is insufficient to resolve many lineages, such as among the basal grade of angiosperms with monosulcate pollen^42^. Tricolpate pollen appears some 25 Ma before the oldest definitive macrofossil eudicots (*Fairlingtonia* from the Potomac Group of Maryland, USA^51^, is known only from vegetative remains; it’s position as a eudicot requires further evidence^42^) (Fig. 3). The gap between the oldest angiosperm pollen (which is monosulcate) and macrofossil is only about 10 Ma (Fig. 3). Gaps are commonly invoked in phylogenomic models of divergence times to explain estimates of angiosperm origins deep into the Mesozoic, even the Triassic^52^, far preceding direct fossil evidence. A general consensus, although, is that stem-group angiosperms may have originated in the Late Jurassic 150–160 Ma, but would have been extremely scarce and ecologically insignificant^42^. Definitive evidence for eudicots first appears well before *Prosphex* was preserved in the Burmese amber, in the latest Barremian to earliest Aptian, based on palynological evidence (possibly even from the mid-Barremian, Isle of Wight^53^); Eudicots are well represented in the late Albian–early Cenomanian of Myanmar based on diverse floral inclusions in the amber^43,44,48,54^.

Prior to and including the time of Burmese amber formation 100 Ma, eight of the ten records of Cretaceous insects with pollen involve gymnosperms (Fig. 3). Moreover, these insects are phylogenetically disparate in five orders and many have structures specialized for feeding on gymnosperm cones, strobili and pollen tubes^8^. A striking pattern is that by the Turonian in the Late Cretaceous—exquisitely preserved as fusainized flowers from the Raritan Formation of New Jersey—there existed a suite of floral features associated with insect pollination: asymmetric and tubular corollas, clawed petals, staminodal nectaries, pollen viscin threads, dioecious flowers, nectary disks, resin glands, and staminal food bodies^55^. Insects probably began their intimate relationship with angiosperms when these plants debuted in the earliest Cretaceous or Late Jurassic; by 90 Ma, their relationship appears to have been consummated.

The two Cretaceous insects found with angiosperm pollen, both in Burmese amber, involve morphologically generalized insects, a permopsocid and *Prosphex*. Likewise, two morphologically generalized, stem-group species also in Burmese amber apparently belong to groups that today are major pollinators: the putative bee *Melittosphex*^56^ and the flower fly *Prosyrphus*^57^. *Melittosphex* is problematic, because its hairs are barely plumose, it lacks the pronotal lobes typical of apoids, and it has a broad pronotum, like chrysidoids. Burmese amber has been especially revealing, because it was formed and preserved in massive quantities;^32^ it will no doubt be yielding much further pollen–insect evidence.

Three main factors contribute to the effectiveness of aculeate wasps as pollinators, which apparently pre-adapted bees to become the predominant pollinators, one being the strong, directed flight of the larger species, particularly ones in the Apoidea and Vespoidea. Another is intelligence. All insects undoubtedly are capable of avoidance learning, but longer-lived species that are active foragers, such as aculeates, are adept at associative learning. Honey-bee foragers, for instance, learn and communicate to hive members the direction, distance, and quality of nectar sources, among various other tasks^58^. Even though sociality is usually associated with keen learning ability, species of solitary, ground-nesting bees, and other wasps, e.g., visually imprint their nest location on a learning flight^59^. Learning allows an individual to specialize as conditions allow; in pollinators, it promotes foraging fidelity and flower constancy^60^. Such intelligence has a neurological basis in an area of the insect brain called the mushroom bodies, which function in the processing of olfactory, gustatory, visual, and tactile information, and associative learning. Mushroom bodies are highly developed in apocritan wasps^61^.

Lastly, the aculeate sting allows wasps to forage exposed on flowers with relative impunity, testament to which are the hundreds of flower-visiting syrphid and conopid flies, beetles, and diurnal moth species that mimic the bold black-and-yellow aposematic color patterns of vespids and bees. Basal lineages of Vespidae existed by the time Burmese amber was formed in the mid-Cretaceous^62,63^ and a species of zhangsolvid fly even exists in Burmese amber with vespid-like aposematic patterns^64^. For *Prosphex*, its body coloration was either uniform or the patterns were not preserved, which is typical of inclusions in amber, the zhangsolvid being a rare exception.

The main radiation of aculeate wasps preceded that of angiosperms by about 30 million years. The oldest direct (fossil) evidence of aculeates is the apparent stem-group family Bethylonymidae, from the Upper Jurassic of Kazakhstan^36^. By the Early Cretaceous, 140–135 Ma, several extinct and extant families of aculeates existed, with the main radiation of families occurring some 150–135 Ma^4^. By the time of the main period of angiosperm radiation, some 120–90 Ma, aculeate wasps were well evolved.

Burmese amber was formed in a dense, megathermal conifer forest at or near the paleoequator, in a wet paleoclimate with organisms typical of modern tropical rain forests: velvet worms (Onycophora), diverse ants, and termites, even dicot leaves with well-developed drip tips^32^. In the understory were diverse herbaceous and shrubby angiosperms, the type of biological community that basal, ANITA-grade angiosperms largely inhabit today^65^. If the early angiosperms were scattered and localized throughout the forest, growing in light gaps and littoral areas edging streams and ponds^65^, they would have required efficient, reliable, and competitive pollen vectors, such as aculeate wasps. Despite the diversity of long-tongued insects in the Early Cretaceous, many of these may actually have been gymnosperm pollinators that did not transition to the pollination of angiosperms. The hypothesis that early angiosperms were visited by myriad small, generalized insects^4,66^ is gathering new supportive evidence. Lastly, the sum of evidence is compelling for entomophily being the ancestral reproductive mode in angiosperms, which may explain a major gap in the pollen fossil record, particularly for the eudicots^42^ (Fig. 3). The rarity of pollen in the Valanginian has traditionally been attributed to the rarity and dispersion of early angiosperms; however, as entomophilous pollen is far less common in geological sediments, it would further obscure the earliest traces of angiosperms.

## Methods

### The amber

Burmese amber derives from the middle of the Cretaceous, approximately near the boundary between the Early and Late Cretaceous (Albian–Cenomanian stages, Fig. 3), ca. 100 million years old based on U-Pb isotope dating^67^. It is the largest and most diverse Cretaceous deposit in the world and is marketed commercially worldwide. The piece of amber studied here, AMNH Bu-KL18-31, was among several hundred pieces that were acquired by the AMNH from Burmese amber dealers. The source of the amber is from outcrops in Kachin Province, northern Myanmar^32^. Similar to most marketed Burmese amber, the piece was a polished cabochen; subsequently, two flat, opposing surfaces were trimmed, ground, and polished on each side of the wasp and pollen plume, to obtain close views of the insect and pollen inclusions (a water-fed diamond-edged trim saw and Buehler Ecomet lapidary wheel were used). The piece was not embedded in synthetic resin in order to minimize thickness for high-magnification work and to optimize CT scan imaging, but it will be embedded in EpoTek 301-2 to preserve the amber against long-term degradation.

### Palynological analysis

Two pollen grains close to the surface of the amber were imaged at the AMNH with a Zeiss LSM710 (AxioObserver) confocal laser-scanning microscope using a Plan-Apochromat ×20/0.8 M27 objective. Fluorescence images (both single and *Z*-stacks) were taken at an excitation wavelength of 488 nm and an emission wavelength of 621 nm, but resolution was insufficient and background fluorescence too bright for useful images, so the pollen grains were studied at the Museo Geominero in Madrid by wide-field transmitted light microscopy, using an Olympus BX51microscope. Photomicrographs were made with a Color View IIIu digital camera attached to the microscope. Morphology of the studied grains were described using established terminology^68,69^. Pollen grains were mapped by hand using a camera lucida attached to an Olympus BX41 microscope. The habitus of the wasp showing the pollen load was photographed using a Canon EOS 650D camera with Macrofotografía software, version 1.1.0.5.

### CT imaging

The amber piece was examined using X-Ray micro-CT at the American Museum of Natural History Microscopy and Imaging Facility, with a GE Phoenix v/tome/x s240 and 180 kV source. An initial scan at 70 kV and 220 μA with 500 ms exposures established baseline parameters and determined approximate internal preservation of the wasp. A total of 1800 images were taken; for each image 6 exposures were used, from which one was skipped and 5 averaged. The specimen was scanned a second time at greater resolution and significantly longer duration. The cuticle of insects in amber occasionally have X-ray absorption values close to that of the amber itself. Longer scans at lower energies can help achieve greater dynamic range, better differentiating internal and external morphology, but this varies greatly with, e.g., inclusion preservation and composition of the amber (e.g., Fig. 1a). As with the first scan, 1800 images were taken, but at lower beam energy and current (60 kV, 200 μA). Exposures were 1000 ms and as with the first scan each image used six exposures with a skip of 1 and average of 5. Three vertically stacked scans were used to reach a smaller final voxel size of approximately 2.8 μm^3^.

Volume reconstruction from raw projections used GE Phoenix datos/x 2.3.2. A combination of manual and semi-automatic geometry correction was used and reconstructed volumes were exported as 16 bit TIFF stacks for post-processing. Three sections comprising the second scan were manually combined using Fiji/ImageJ 2.0.0^70,71^ and 3D Stitching^72^. Volume datasets were exported in NRRD format before segmentation and rendering. Post-processing and isolation of regions of interest via segmentation used the open-source project 3D Slicer (www.slicer.org)^73^. As the Slicer project is under continuous development, various nightly builds were used, spanning versions 4.7 through 4.11. Segmentation was done with the Segment Editor module, primarily using a combination of thresholding and hand selection of areas of interest. Visualizations were rendered using either 3D Slicer or Blender 2.78c (Blender Foundation) with the Cycles render engine. Differences between a high-density mass in the buccal cavity and the surrounding region were studied by isolating the head capsule as a separate volume dataset, for plotting an intensity histogram. Segmentation used thresholding and was rendered using transparent shaders to illustrate the size, granularity, and location inside the mouthparts.

### Reporting summary

Further information on research design is available in the Nature Research Reporting Summary linked to this article.

## Supplementary information

Supplementary Information

Reporting Summary

## Data Availability

CT scan raw files are deposited in and available publicly at https://datadryad.org/stash/share/sSWP3uN9c_08EoYeozfnL_e2usagyA9RhW6FlhQtA-w. The holotype specimen of *P. anthophilos* is housed at the American Museum of Natural History (Division of Invertebrate Zoology), its collections of which are available for study to qualified researchers.
