# Supplementary material for: Direct evidence for eudicot pollen-feeding in a Cretaceous stinging wasp (Angiospermae; Hymenoptera, Aculeata) preserved in Burmese amber
Source: Commun Biol. 2019 Nov 7;2:408. doi: 10.1038/s42003-019-0652-7 (PMC6838090; doi:10.1038/s42003-019-0652-7)
Supplement: Supplementary file 1 — Supplementary Information [file 42003_2019_652_MOESM1_ESM.pdf]

Supplementary Information

Supplementary Figures

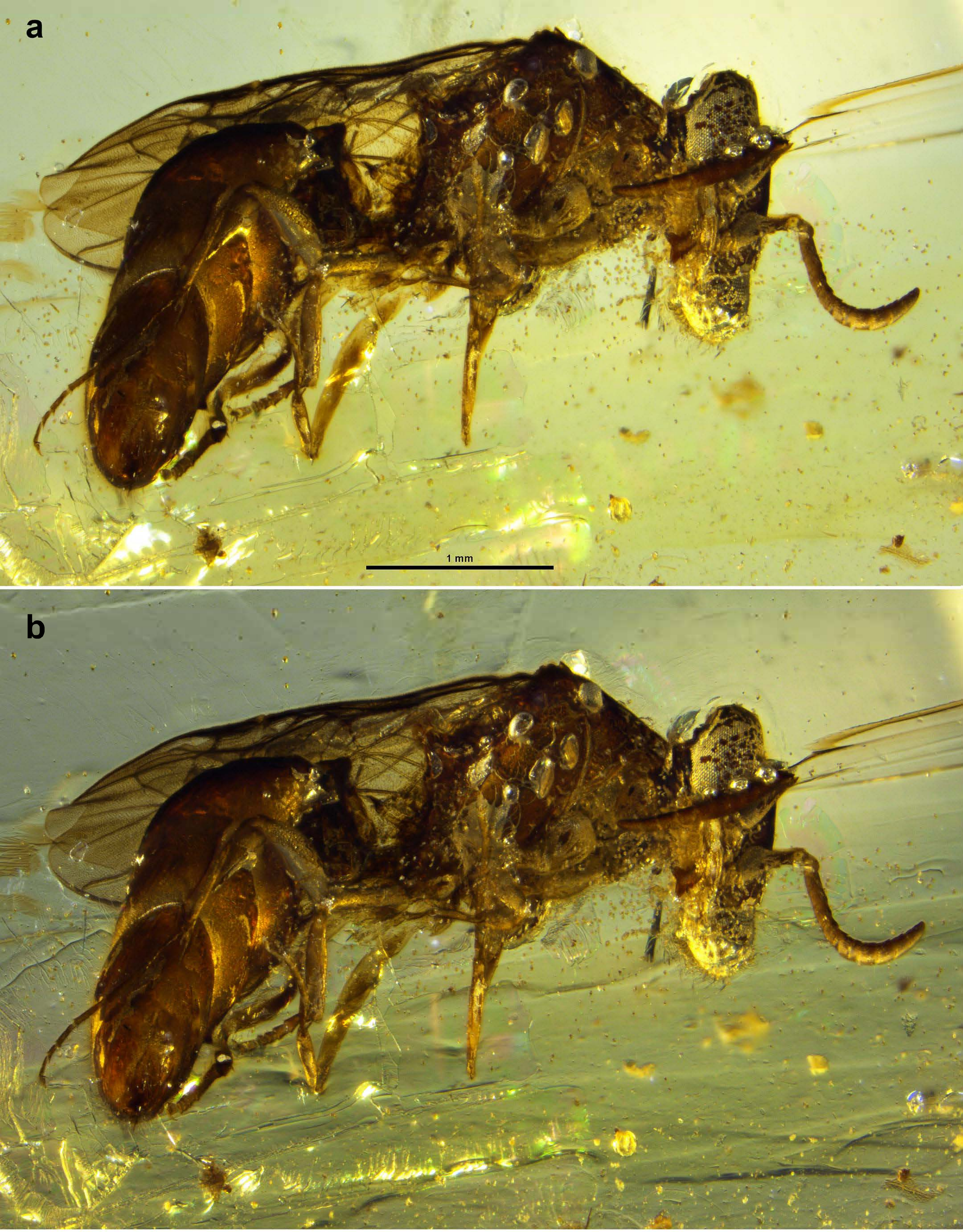

Supplementary figure 1: *Prosphex anthophilos,* Holotype (AMNH Bu-KL18-31). A. Ventrolateral view of habitus, with full backlighting. B. Ventrolateral view (same magnification as A, scale line 1.0 mm), with partial backlighting and partial reflected (refracted) light, which accentuates flow and stress lines and fine fractures within the piece.

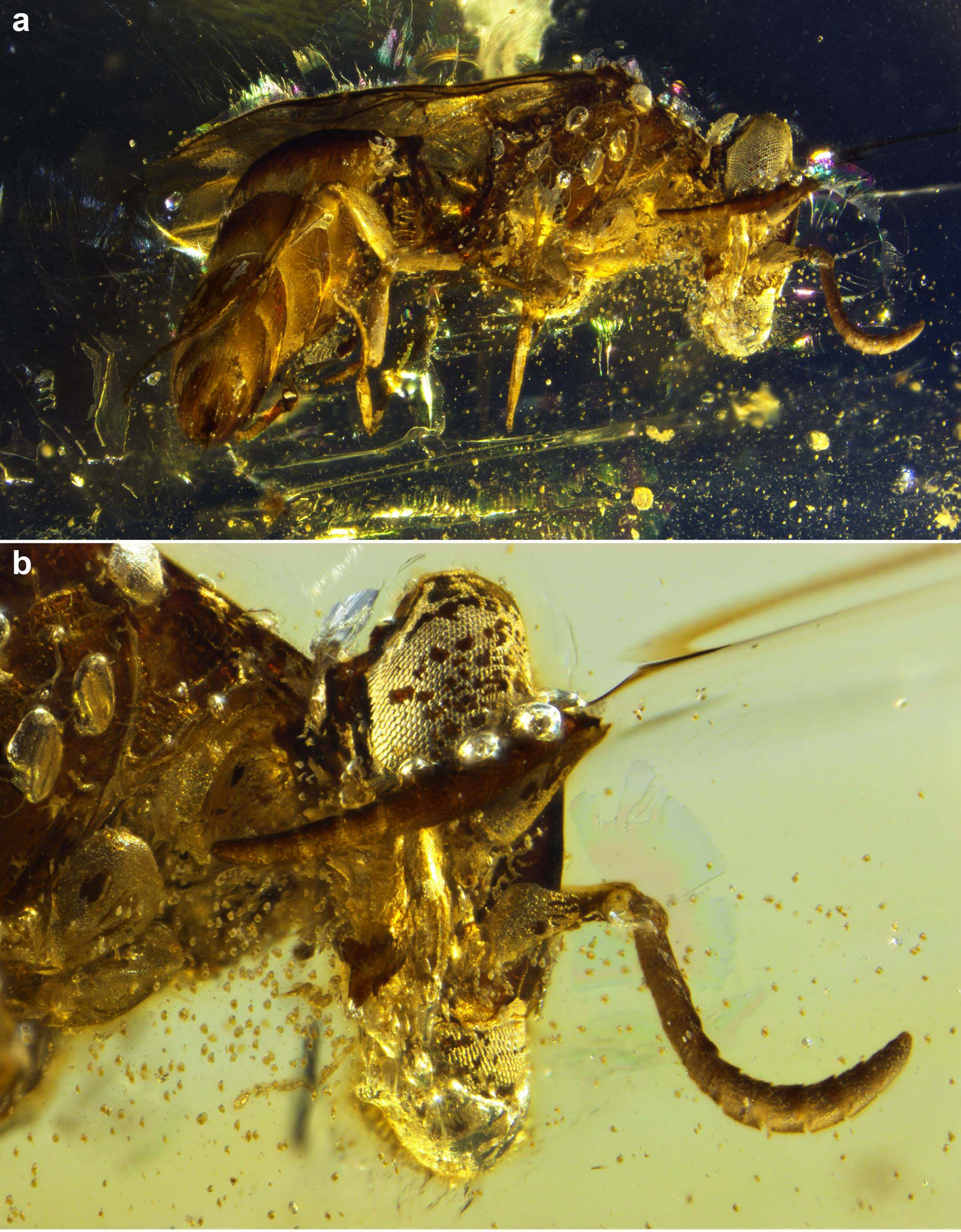

Supplementary figure 2: *Prosphex anthophilos* (AMNH Bu-KL1 8-31). A. Ventrolateral view of habitus under darkfield illumina-tion. B. Detail of head, ventrolateral view with reflected light, showing details of prothorax, antennal segmentation, mandibles (crossed, dark structures in center), and pollen on and near the head.

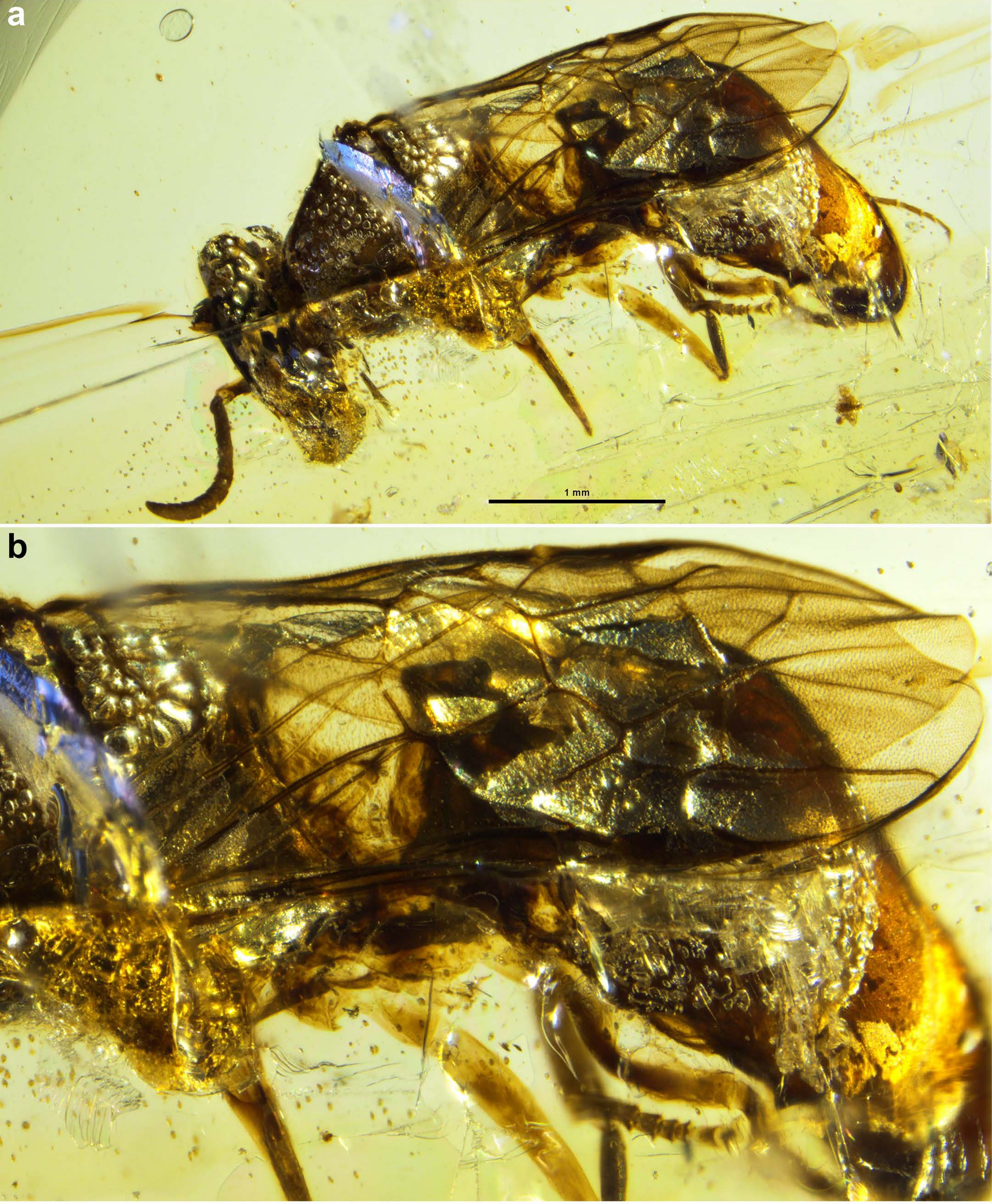

Supplementary figure 3: *Prosphex anthophilos* (AMNH Bu-KL18-31). A. Dorsolateral view under reflected light, showing fine bub-bles covering some of the thorax. Scale line 1.0 mm. B. Detail of waist area, anterior portion of the metasoma, and especially the wings. The wings are overlapping, so venation is difficult to resolve. The veins were too fine to resolve using CT scanning, so the venation was reconstructed using direct observation under stereomicroscopy at magnifications of 80-1 l 6X. A drawing tube was used to make an initial sketch of the venation, which was then refined and details added by examining the wing at different focal planes and by slight tilting of the specimen at various angles in order to resolve overlapping veins.

Supplementary Notes, Discussion, Methods

***Prosphex anthophilos* Grimaldi and Engel, new species**
ZooBank LSID (genus): urn:lsid:zoobank.org:act:1F27660C-5025-479E-8C4F-F7C10600EF24
ZooBank LSID (species): urn:lsid:zoobank.org:act:8F253B89-29D8-4FE7-B231-2A0899A4448D

Description: Head: Hypognathous, short, broad, with broad frontal surface, head width 1.45 mm, head length 0.60 mm; compound eyes widely separated, not emarginate, inner ocular margins comparatively straight (Figs. 1a, g). Ocelli apparently lacking; frons slightly, finely punctate. Antennae relatively short (ca. 1.5 mm); situated very low on face, meeting epistomal sulcus (Fig. 1g; Supplementary Figure 2b), minute crescentic collar around medial margin of torulus; radicles exposed, separated by distance approximately equal to their diameter; antenna with 13 articles: pedicel large, length approximately equal to that of flagellomeres 1–5, width 1.5× that of flagellomeres (broadest at base); pedicel shorter than all flagellomeres; flagellomeres short, length slightly more than width for only apical three (flagellomeres 9–11) (Figs. 1a, g; Supplementary Figure 2b), with very faint, minute, ovoid plaque sensilla on middle articles; tyloids absent. Subantennal area and sulci absent. Clypeus short, broad, bare; margin simple. Mandibles symmetrical or nearly so, roughly long isoscelean in shape; malar edge serrate, with five equal-sized teeth (apical one slightly larger), anterior surface with scattered fine setulae. Palpi partially exposed: four maxillary palpomeres, three labial; length of exposed basal maxillary palpomere equal to length of apical three palpomeres, apical maxillary palpomere slightly drop-shaped; labial palpus much shorter, apical palpomere pointed (Fig. 1g). Occiput broad, flat, with narrow but well-developed occipital carina encompassing ca. 0.5× total head width; gena not protruding (Fig. 1f).

Mesosoma: Short, compact (length 1.5 mm, metasomal length 2.2 mm), broad; with virtually no pilosity; propodeum steep (Figs. 1a, g; Supplementary Figures 1a, 3a). Pronotum: in lateral view large, triangular, ventral corner with 3–4 very faint foveae; in dorsal view a narrow bridge. Propleuron and prosternum apparently highly integrated anteriorly. Mesopleuron largely comprised of mesepisternum; metapleuron if present unapparent, scrobal sulcus unapparent; mesepisternum with faint, shallow foveae on dorsal half, none ventrally; sternaulus not present (Figs. 1a, g). Metapleuron narrow, ill-defined posteriorly. Mesoscutum large, no cuticular sculpturing or foveae, highly integrated with sutures reduced: median and admedian lines absent; notauli apparently absent, parapsidal lines faint, evanescent anteriorly, ca. 0.35× length of mesoscutum, widely separated (Fig. 1f). Mesoscutellum relatively small, length ca. 0.4× that of mesoscutum, width 0.6×, lateral surface with shallow fovea. Axilla small, shallow. Metanotum very small (Fig. 1f). Propodeum broad, abruptly sloped, with two pairs of spines [this area compressed and distorted, so structure of spines and cuticular surface of propodeum not preserved, including development of metapostnotum]; propodeal spiracle well-developed (Fig. 1g); petiole very short such that metasoma is largely sessile.

Legs: Short, relatively slender, femora not at all crassate, tarsi 5-merous; coxae short, stout, ones in each pair abutting medially; trochantellus absent; mesofemur with faint basal ring; probasitarus with calcar (ventroproximal surface slightly emarginate, spur with short, faint feathering at base); apical tibial spurs 1:2:2, protibial one very long, length 4× width of tibia, metatibial spurs simple (including inner one); tarsomeres without plantar lobes; claws with preapical ventral tooth; arolium well developed (Fig. 1g).

Wings: Forewing relatively broad, length 2.7 mm, greatest width 1.0 mm; with 14 well-defined cells, their configurations and identities provided in Fig. 1d. Bullae present in five crossveins, as indicated in Fig. 1d. Apices of M and CuA1 evanescent, not quite reaching apical and subapical margin of wing. Hind wing (Fig. 1e): Only partially visible (mostly obscured under forewing), cells R, M+CuA partially defined; all veins except R+Sc tubular only, not sclerotized; hamuli not observable.

Metasoma: Relatively short; petiole short (detailed structure obscure); no postpetiole, no constriction between metasomal segments (Figs. 1a, g; Supplementary Figures 1–3). Lateral edges of terga and sterna meet laterally in a mid-lateral line, not overlapping (preservational, owing to postmortem expansion of metasoma?); tergum 2 largest. Number and position of spiracles not observable. Sting well developed (Fig. 1h), much of it retracted into metasoma based on CT scans; everted portion with a very thin dorsal sheath at base that is open ventrally (probably the dorsal valve, or gonoplac) (Fig. 1h). Cerci absent.

In order to provide more detailed photomicrographic data on the wasp, high resolution images of the specimen were prepared at the AMNH under different magnifications and lighting. A Nikon SMZ1500 stereoscope with a transmitted light base was used along with four gooseneck fiber optic lights (Schott KL1600 LED) for reflected and transmitted light. Photos were taken with a Nikon Digital Sight DS-Ri1 camera on the stereoscope, using Nikon Elements® software version D4.30.01. Photos were taken at the highest resolution (4076 × 3116); multiple focal planes were Z-stacked using Elements, and the final image slightly sharpened electronically. Photos of the wasp habitus were taken with fully transmitted light; with partially transmitted (partially diffracted) light, to accentuate flows and fractures; and with darkfield (reflected light only).
