# Supplementary material for: Direct evidence for eudicot pollen-feeding in a Cretaceous stinging wasp (Angiospermae; Hymenoptera, Aculeata) preserved in Burmese amber
Source: Commun Biol. 2019 Nov 7;2:408. doi: 10.1038/s42003-019-0652-7 (PMC6838090; doi:10.1038/s42003-019-0652-7)
Supplement: Supplementary file 2 — Reporting Summary [file 42003_2019_652_MOESM2_ESM.pdf]

nature research

Corresponding author(s): David A. Grimaldi

Last updated by author(s): Aug 27, 2019

Reporting Summary

Nature Research wishes to improve the reproducibility of the work that we publish. This form provides structure for consistency and transparency in reporting. For further information on Nature Research policies, see Authors & Referees and the Editorial Policy Checklist.

Statistics

For all statistical analyses, confirm that the following items are present in the figure legend, table legend, main text, or Methods section.

n/a	Confirmed	
☒	☐	The exact sample size (*n*) for each experimental group/condition, given as a discrete number and unit of measurement
☒	☐	A statement on whether measurements were taken from distinct samples or whether the same sample was measured repeatedly
☒	☐	The statistical test(s) used AND whether they are one- or two-sided<br>*Only common tests should be described solely by name; describe more complex techniques in the Methods section.*
☒	☐	A description of all covariates tested
☒	☐	A description of any assumptions or corrections, such as tests of normality and adjustment for multiple comparisons
☒	☐	A full description of the statistical parameters including central tendency (e.g. means) or other basic estimates (e.g. regression coefficient) AND variation (e.g. standard deviation) or associated estimates of uncertainty (e.g. confidence intervals)
☒	☐	For null hypothesis testing, the test statistic (e.g. *F*, *t*, *r*) with confidence intervals, effect sizes, degrees of freedom and *P* value noted<br>*Give P values as exact values whenever suitable.*
☒	☐	For Bayesian analysis, information on the choice of priors and Markov chain Monte Carlo settings
☒	☐	For hierarchical and complex designs, identification of the appropriate level for tests and full reporting of outcomes
☒	☐	Estimates of effect sizes (e.g. Cohen's *d*, Pearson's *r*), indicating how they were calculated

Our web collection on statistics for biologists contains articles on many of the points above.

Software and code

Policy information about availability of computer code

Data collection	no computer code, commercial or custom, was used in data collection. Only publicly available software which is cited and referenced in the ms..
Data analysis	no computer code, commercial or custom, was used in data analysis.

For manuscripts utilizing custom algorithms or software that are central to the research but not yet described in published literature, software must be made available to editors/reviewers. We strongly encourage code deposition in a community repository (e.g. GitHub). See the Nature Research guidelines for submitting code & software for further information.

Data

Policy information about availability of data

All manuscripts must include a data availability statement. This statement should provide the following information, where applicable:

- Accession codes, unique identifiers, or web links for publicly available datasets
- A list of figures that have associated raw data
- A description of any restrictions on data availability

The raw (.tiff) CT scan files (ca. 1.5 GB) are uploaded to Dryad for public availability; a link to the data will be provided in the ms proof.

Field-specific reporting

Please select the one below that is the best fit for your research. If you are not sure, read the appropriate sections before making your selection.

☐ Life sciences ☐ Behavioural & social sciences ☒ Ecological, evolutionary & environmental sciences

Ecological, evolutionary & environmental sciences study design

All studies must disclose on these points even when the disclosure is negative.

Study description	Grimaldi et al. report on a primitive stinging wasp preserved in mid-Cretaceous amber from Myanmar, encapsulated just after it fed on the pollen of a eudicot. Micro-CT reveals a pollen mass in the mouth cavity of the wasp, supporting the concept that generalized insect anthophiles were important pollinators of early angiosperms.
Research sample	The study is based on a unique specimen of a wasp and associated pollen grains preserved in a piece of amber.
Sampling strategy	N/A
Data collection	Data collection is described in methods: light microscopy (both compound [transmitted] and stereo [reflected]), and micro-CT scanning.
Timing and spatial scale	N/A
Data exclusions	N/A
Reproducibility	The micro-CT raw .tiff files will be publicly available for reconstruction by others; the specimen is available for study to qualified researchers, in keeping with policy of the American Museum of Natural History (where the specimen is housed).
Randomization	N/A.
Blinding	N/A

Did the study involve field work? ☐ Yes ☒ No

Reporting for specific materials, systems and methods

We require information from authors about some types of materials, experimental systems and methods used in many studies. Here, indicate whether each material, system or method listed is relevant to your study. If you are not sure if a list item applies to your research, read the appropriate section before selecting a response.

Materials & experimental systems

n/a	Involved in the study	
☒	☐	Antibodies
☒	☐	Eukaryotic cell lines
☐	☒	Palaeontology
☒	☐	Animals and other organisms
☒	☐	Human research participants
☒	☐	Clinical data

Methods

n/a	Involved in the study	
☒	☐	ChIP-seq
☒	☐	Flow cytometry
☒	☐	MRI-based neuroimaging

Palaeontology

Specimen provenance	The specimen was collected about 5 years ago by Burmese amber miners in Kachin Province, northern Myanmar, purchased soon thereafter by a private individual and donated to the AMNH. Burmese amber has been marketed/sold commercially by Burmese amber dealers for many years.
Specimen deposition	The unique specimen (the holotype) is deposited in the amber fossil collection, Division of Invertebrate Zoology, American Museum of Natural History, NY NY 10024.
Dating methods	The date of age applied, ca. 100 Ma, is based on U-Pb isotope dating of zircons in the surrounding sediments of the amber deposits, as published previously by Shi et al and cited in the manuscript.

☒ Tick this box to confirm that the raw and calibrated dates are available in the paper or in Supplementary Information.
